# Emotional Intelligence and Academic Performance among Male and Female Medical Undergraduates in rural Sindh province, Pakistan

**DOI:** 10.12669/pjms.42.6.14634

**Published:** 2026-06

**Authors:** Seema Bibi Qureshi, Ambreen Usmani, Junaid Tariq

**Affiliations:** 1 Seema Bibi Qureshi, Professor and HOD, Department of Obstetrics and Gynecology, Suleman Roshan Medical College Tando Adam, Sindh, Pakistan; 2 Ambreen Usmani, Principal (SMC) & Professor of Anatomy, Sindh Medical College (SMC), Jinnah Sindh Medical University (JSMU), Karachi, Pakistan; 3 Junaid Tariq, Senior Program Coordinator & Research Facilitator, Department of Medical Education, College of Physicians and Surgeons, Karachi, Pakistan

**Keywords:** Academic Performance, Emotional Intelligence, Pakistan, Medical, Male and female Students

## Abstract

**Background & Objectives:**

Medical undergraduates face demanding requirements that heighten risk of stress, burnout, and poor performance. Emotional intelligence (EI), the ability to perceive, understand, and manage emotions supports resilience, well-being, and learning. However, evidence from Pakistan is inconsistent, with existing studies generally reporting moderate EI levels and context-dependent associations with academic outcomes. Our objective was to assess the emotional intelligence levels of medical undergraduates, compare EI between male and female students, and examine the association of EI with academic performance.

**Methodology:**

A cross-sectional study was conducted from 1^st^ January 2024 to 31^st^ December 2024 among 172 MBBS students (years 2–4) at Suleiman Roshan Medical College, Tando Adam, Sindh. Data was collected using a structured questionnaire covering demographics, academics, and the Trait Emotional Intelligence Questionnaire–Short Form (TEIQue-SF). Academic performance was assessed by cumulative grade point average (CGPA). Analyses included descriptive statistics, independent-samples t-tests for gender, and Pearson’s correlations for associations between EI and academic outcomes.

**Results:**

Students demonstrated moderate EI (global EI mean = 4.53, SD = 0.77). Well-being was the strongest factor (mean = 5.16, SD = 0.94), and self-control the weakest (mean = 4.03, SD = 1.04). No significant differences among male and female students were found across global EI or its factors (p > 0.05). Reliability analysis showed good internal consistency (Cronbach’s α = 0.88 for global EI; subscales ranged from 0.76 to 0.84). EI factors showed no significant link with academic performance, except for a weak positive correlation between sociability and CGPA (r = 0.153, p = 0.046), reflecting only a weak effect size.

**Conclusion:**

Medical students showed moderate levels of emotional intelligence, with no significant differences between male and female groups. Emotional intelligence was generally unrelated to academic performance, except for a weak positive association between sociability and CGPA.

## INTRODUCTION

Medical education is a demanding training pathway that exposes students to academic, financial, social, and psychosomatic challenges. Stress, anxiety, and burnout are common among medical students.^1^ Female students may additionally encounter socio-cultural barriers such as household responsibilities, discrimination, and harassment.^2^ Emotional intelligence (EI), defined by Salovey and Mayer in 1990 as “the ability to monitor one’s own and others’ feelings and emotions, to discriminate among them and to use this information to guide one’s thinking and actions,” has been proposed as a novel factor that may help students cope with these pressures.^3^

EI is studied through two main models i.e. ability and trait. Ability EI is linked to actual emotional skills and is measured through objective performance tests while trait EI reflect self-perceptions of emotional strengths and measured subjectively via self-reported questionnaires. Trait EI has shown stronger and persistent criterion validity in educational contexts and is commonly measured using tools such as the TEIQue, EQ-i, and SREIT.^4^,^5^ The TEIQue-SF provides a broad assessment of global EI and its four factors: well-being, self-control, emotionality, and sociability.

Global evidence on the role of EI in academic success is varied. Some meta-analyses report positive associations between EI and academic achievement, while others find only weak or inconsistent correlations’.^6^,^7^ In Pakistani context, studies from Punjab have suggested positive links^8^, but other local and regional studies have failed to establish significant relationships’.^9^,^10^ Inconsistencies may stem from differences in EI measurement tools, variations in definitions of achievement, and sample characteristics like discipline and year of study.^11^ Importantly, most existing evidence is urban-centric and often based on general student populations, leaving a critical evidence gap regarding medical undergraduates in rural colleges where socio-cultural and educational environments differ markedly.^12^ Moreover, it remains unclear whether EI contributes independently to student outcomes within Pakistan’s exam-driven educational system, which prioritizes passing examinations and attaining high CGPAs over fostering professional competence and intellectual development, the domains that inherently depend on emotional intelligence.

The objectives of this study are to assess EI levels among medical students in rural Sindh, compare scores between male and female groups, and investigate linkage between EI and academic performance. The rationale is to generate locally relevant evidence on whether EI should be incorporated into medical and pre-medical curricula to support wellbeing and professional development, rather than being viewed solely as a predictor of exam success.

## METHODOLOGY

This cross-sectional quantitative study was conducted from 1st January to 31st December 2024 at Suleiman Roshan Medical College (SRMC), Tando Adam, Sindh, Pakistan, a private institution enrolling student in accordance with Pakistan Medical and Dental Council regulations. The study population comprised MBBS students in their 2nd to 4th academic years. Eligibility requirement includes enrollment in the MBBS program and appearance in their last annual examinations. First-year, final-year, and non-MBBS students (e.g., physiotherapy, nursing) were excluded.

### Sample size and sampling:

Sample size was determined using the Raosoft sample size calculator, with a population of approximately 300 students (100 per batch), a 95% confidence interval, and a 5% margin of error. The required sample was 172 students. A non-probability convenience sampling approach was employed

### Data collection instruments:

Data were collected using a structured questionnaire comprising two sections:

***1. Demographic and academic Information:*** Included age, sex(male/female), academic year, and the most recent cumulative grade point average (CGPA) from annual examinations. CGPA was selected as the key academic outcome because it provides a consistent and comprehensive reflection of students’ overall academic performance.

***2. Emotional Intelligence:*** Measured using the Trait Emotional Intelligence Questionnaire –Short Form (TEIQue-SF). This 30-item tool is derived from the full TEIQue and assesses global trait EI as well as four factors consisting of well-being, self-control, emotionality, and sociability. Each factor was rated on a 7-point Likert scale (1 = completely disagree to 7 = completely agree). The TEIQue-SF has been validated in diverse student populations and demonstrates acceptable reliability and construct validity.^13^,^14^ Internal reliability of the TEIQue-SF for this sample was also calculated using cronbach’s alpha for global EI and its subscales.

EI scores were obtained using the TEIQue-SF, following the standardized scoring guidelines provided by the instrument.^15^ In our study, student responses with average scores between 1.0 and 2.99 were categorized as Low, scores from 3.0 to 4.99 as Moderate, and scores from 5.0 to 7.0 as High, following the approach described by Mohzan et al.^16^

### Data collection procedure:

The students were approached in person and given a brief explanation of the study’s purpose and instructions for properly filling out the questionnaire. They were informed that participation was voluntary and confidential. The questionnaire, along with an informed consent form, was uploaded via Google Forms, and the link was distributed through WhatsApp.

### Data analysis method:

Data was entered and analyzed using SPSS version 22. Descriptive statistics (means, standard deviations, and frequencies) were used to summarize demographic and EI data.

### Objective 1 (EI Levels):

Mean and standard deviation of EI scores (global and 4 trait factors) were calculated for all participants.

### Objective 2 (Differences between male and female students):

Independent samples *t*-tests were conducted to compare EI scores between male and female students.

### Objective 3 (Association with Academic Performance):

Pearson’s correlation coefficient was used to examine the relationship between EI scores and CGPA. Where appropriate, Chi-square tests were applied for categorical comparisons. A *p*-value < 0.05 was considered statistically significant.

### Ethical considerations:

Ethical approval was obtained from the institutional review board of SRMC vide reference number SRMC/PRINCIPAL/367; Dated: December 13, 2023. Participation was voluntary, and students could withdraw at any stage. Informed consent was obtained electronically before data collection. All responses were kept confidential, and results were reported in aggregate form only.

## RESULTS

Of the 172 participants, 97 (56.4%) were female and 75 (43.6%) were males. The majority were aged 21–23 years (n=128,74.4%), followed by 24–26 years (25,14.5%), 18–20 years (18,10.5%), and >26 years (1,0.6%). Among the included participants (n = 172), the majority had appeared in 4th year (n = 114, 66.3%), followed by 3rd year (n = 34, 19.8%) and 2nd year (n = 24, 14.0%). Regarding academic performance, Mean examination marks were 71.8 (SD ± 6.5, range 51–88), and mean CGPA was 3.30 (SD ± 0.38, range 2.0–4.0).

Descriptive statistics of EI levels are presented in Table-I. Overall, participants demonstrated moderate self-perception of emotional capability. Among the four core factors, well-being scored highest (M = 5.16 ± 0.94), while self-control scored lowest (M = 4.03 ± 1.04).” Reliability analysis confirmed good internal consistency of the TEIQue-SF in this cohort (α = 0.88 for global EI; subscales α = 0.76–0.84).

**Table-I:** Descriptive Statistics of Emotional Intelligence Factors (n = 172).

EI Factor	Mean	Standard deviation	Min	Max
Global EI	4.53	0.77	2.33	6.43
Well-being	5.16	0.94	2.00	7.00
Self-control	4.03	1.04	1.17	6.67
Emotionality	4.47	1.03	1.50	7.00
Sociability	4.28	0.98	1.33	7.00

EI scores across male and female student groups were presented in Table-II. No significant differences were found in global EI or its four factors (p > 0.05). Males and females scored similarly in well-being, self-control, emotionality, and sociability. Reported p-values, Cohen’s d, and 95% confidence intervals all indicate negligible differences, with small effect sizes (d < 0.20) and confidence intervals crossing zero.

**Table-II:** Gender Differences in EI factors (n=172).

EI Factor	Male (M ± SD)	Female (M ± SD)	t	p-value
Global EI	4.56 ± 0.71	4.50 ± 0.81	0.52	0.602
Well-being	5.04 ± 1.03	5.24 ± 0.85	-1.34	0.182
Self-control	4.09 ± 1.02	3.99 ± 1.06	0.66	0.510
Emotionality	4.52 ± 0.99	4.43 ± 1.07	0.55	0.584

Table-III shows correlations between EI factors and CGPA. No significant correlations were found for global EI or its factors, except sociability, which showed a weak positive association with CGPA (r = 0.153, p = 0.046). Figure. A illustrates this trend with a regression line and wide 95% confidence band, highlighting its meek and uncertain nature. Given that multiple correlations were tested between global EI and its four factors with CGPA, the possibility of Type-I error cannot be excluded. As no formal correction (e.g., Bonferroni adjustment) was applied, the observed association should therefore be interpreted with caution.

**Table-III:** Pearson’s Correlations between EI factors Scores and cumulative grade point average (CGPA) n=172.

EI Factor	r (Correlation Coefficient	P value
Global EI	0.075	0.330
Well-being	0.100	0.191
Self-control	-0.023	0.762
Emotionality	0.004	0.960
Sociability	0.153	0.046*

**Fig.1:**
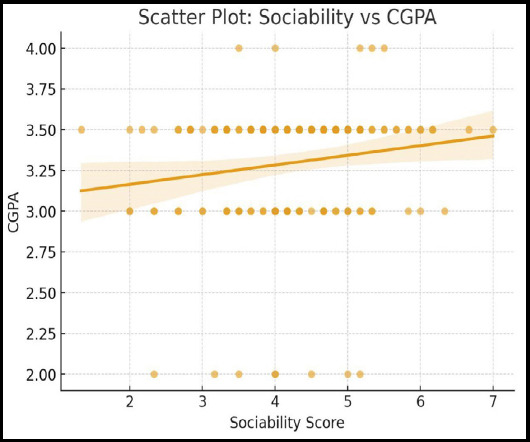
Scatter Plot: Sociability vs. CGPA.

## DISCUSSION

This study assessed emotional intelligence (EI) levels of medical undergraduates across all factors. Students demonstrated moderate global EI scores (M = 4.53, SD = 0.77), reflecting balanced but not high emotional functioning. Such levels suggest adequate adaptability and motivation, qualities that are theoretically important for coping and teamwork in medical education, though the present dataset revealed only minimal empirical association with CGPA. Among the EI factors, well-being scored highest (M = 5.16), indicating self-perceptions of optimism, happiness, and self-esteem. While these attributes are conceptually linked to resilience and healthy coping in demanding environments, their direct impact on academic achievement was not assessed in this study.

In contrast, self-control was the lowest scoring factor (M = 4.03, SD = 1.04). Although its association with academic success cannot be inferred from CGPA, it may reflect challenges in regulating emotions under pressure, particularly during high-stakes clinical tasks such as OSCEs or viva examinations. The findings also highlight the potential role of mentorship in helping students manage these challenges. Institutions could offer stress-management and emotional regulation programs to build these skills, supporting students’ well-being and professional development rather than focusing on grades.^17^ Future studies may be planned to explore the causes and impact of low self-control among medical undergraduates.

In this cohort, most EI factors did not show a significant relationship with academic performance (CGPA). This lack of association may reflect cultural and educational contexts in Pakistan, where structured learning and exam-oriented systems reduce the impact of emotional traits such as self-control or emotionality on grades. Yasmin et al. similarly reported that exam-driven environments undermine students’ emotional intelligence and motivation, limiting the application of emotional skills in academic settings.^18^ Therefore, the EI’s relevance in medical education may lie more in fostering professionalism, communication, mentorship, and stress management than in predicting exam performance.

Although the observed association between sociability and CGPA was weak, it nonetheless suggests a potential link worth exploring. Future research employing larger samples, longitudinal designs, and more robust methodologies is recommended to clarify and validate this relationship.

The linkage between male and female students’ academic performance has been proposed globally, our findings do not support this association, consistent with a recent systematic review of 13,909 participants that refuted the role of sex in academic outcomes.^19^ The absence of differences in this cohort may be explained by contextual influences such as variations in educational settings and assessment methods, reliance on trait EI self-report measures that are prone to social desirability bias. Furthermore, cultural expectations in rural Sindh shape how students perceive and report emotional abilities.^20^ In addition, equal access to faculty mentoring and standardized assessments at the institution might have reduced any gaps between male and female students.

Our findings should be interpreted within the context of a newly established rural medical institution, where socio-cultural challenges, limited resources, and exam-driven pressures shape students’ performance and emotional experiences. These contextual factors enrich the interpretation of results, while internationally the study contributes to understanding how cultural and institutional environments influence the role of EI in medical education.

### Recommendations:

Emotional intelligence strategies should be integrated early into the medical curriculum to help students manage academic and clinical stress. Targeted interventions such as stress management training, reflection, mentorship, and peer support can strengthen self-control and emotional regulation. As no gender differences were observed, EI programs should be inclusive. Future research should use multi-institutional longitudinal designs, include both self-report and ability-based EI measures, and assess broader outcomes such as teamwork, communication, burnout, and engagement.

### Strengths:

Strengths include a validated EI tool, representation across academic years, and context-specific insights from rural Sindh with gender data.

### Limitations:

Limitations include the single-institution, cross-sectional design, small sample size, reliance on convenience sampling, and self-reported EI measures alongside traditional academic metrics, which may explain the non-significant associations. Voluntary participation introduces selection bias, so findings may not fully represent all students. Multiple correlations were tested without formal correction, raising the possibility of Type-I error; thus, the weak sociability–CGPA link should be interpreted with caution.

## CONCLUSION

Medical students demonstrated moderate emotional intelligence, with well-being emerging as the strongest dimension and self-control the weakest. No significant gender differences were observed, and EI showed no meaningful association with academic performance apart from a very weak positive correlation with sociability.

### Author’s contribution:

**SBQ:** Conceived and designed the study, drafted the manuscript, and is accountable for its integrity.

**AU:** Provided guidance in study design and critically reviewed the final manuscript.

**JT:** Assisted in developing the data collection tools and performed statistical analysis.
